# The Mode of Inhibitor Binding to Peptidyl-tRNA Hydrolase: Binding Studies and Structure Determination of Unbound and Bound Peptidyl-tRNA Hydrolase from *Acinetobacter baumannii*

**DOI:** 10.1371/journal.pone.0067547

**Published:** 2013-07-03

**Authors:** Sanket Kaushik, Nagendra Singh, Shavait Yamini, Avinash Singh, Mau Sinha, Ashish Arora, Punit Kaur, Sujata Sharma, Tej P. Singh

**Affiliations:** 1 Department of Biophysics, All India Institute of Medical Sciences, New Delhi, India; 2 Central Drug Research Institute, Lucknow, India; NCI-Frederick, United States of America

## Abstract

The incidences of infections caused by an aerobic Gram-negative bacterium, *Acinetobacter baumannii* are very common in hospital environments. It usually causes soft tissue infections including urinary tract infections and pneumonia. It is difficult to treat due to acquired resistance to available antibiotics is well known. In order to design specific inhibitors against one of the important enzymes, peptidyl-tRNA hydrolase from *Acinetobacter baumannii*, we have determined its three-dimensional structure. Peptidyl-tRNA hydrolase (*Ab*Pth) is involved in recycling of peptidyl-tRNAs which are produced in the cell as a result of premature termination of translation process. We have also determined the structures of two complexes of *Ab*Pth with cytidine and uridine. *Ab*Pth was cloned, expressed and crystallized in unbound and in two bound states with cytidine and uridine. The binding studies carried out using fluorescence spectroscopic and surface plasmon resonance techniques revealed that both cytidine and uridine bound to *Ab*Pth at nanomolar concentrations. The structure determinations of the complexes revealed that both ligands were located in the active site cleft of *Ab*Pth. The introduction of ligands to *Ab*Pth caused a significant widening of the entrance gate to the active site region and in the process of binding, it expelled several water molecules from the active site. As a result of interactions with protein atoms, the ligands caused conformational changes in several residues to attain the induced tight fittings. Such a binding capability of this protein makes it a versatile molecule for hydrolysis of peptidyl-tRNAs having variable peptide sequences. These are the first studies that revealed the mode of inhibitor binding in Peptidyl-tRNA hydrolases which will facilitate the structure based ligand design.

## Introduction

Peptidyl-tRNA hydrolase (Pth) is involved in the processing of prematurely dissociated peptidyl-tRNAs from ribosome during protein biosynthesis into peptide and tRNA [1], [2]. Pth acts upon the ester link in peptidyl-tRNA between the peptide and the 2′ or 3′ OH group at the 3′ end of tRNA [3], [4]. The products of this reaction are reused in the cell in the ongoing process of protein biosynthesis in bacteria [5], [6]. The *Acinetobacter baumannii* is an aerobic Gram-negative bacterium commonly found in hospital environments [7]–[10]. Therefore, many hospital patients have been found to suffer from infections caused by *Acinetobacter baumannii* [11]. The bacterium *Acinetobacter baumannii* is responsible for a number of diseases such as pneumonia [12], meningitis [13], septicemia, and urinary [14] and respiratory tract infections [15]–[17]. It has also been reported that the *Acinetobacter baumannii* is increasingly becoming resistant to antibiotics [18**]**–[21]. Since Pth from *Acinetobacter baumannii* (*Ab*Pth) plays an essential role in the survival of bacteria, the inhibition of its function will lead to the death of *Acinetobacter baumannii*. Therefore, the design of tight inhibitors of *Ab*Pth will help in developing useful antibacterial drugs against infections caused by *Acinetobacter baumannii*. In this regard, it is fortunate that the structures of eukaryotic and prokaryotic peptidyl-tRNA hydrolases are not similar [6]. Thus the design of potent inhibitors of prokaryotic peptidyl-tRNA hydrolases will be a good approach.

In order to carry out structure based drug design using *Ab*Pth as the target protein, it is essential to obtain the three-dimensional structure of *Ab*Pth. So far, structures of three other prokaryotic peptidyl-tRNA hydrolases from *Escherichia coli* (*Ec*Pth) [4], *Mycobacterium tuberculosis* (*Mt*Pth) [22] and *Mycobacterium smegmatis* (*Ms*Pth) [23] are available. Recently, structure of an unbound peptidyl-tRNA hydrolase from *Pseudomonas aeruginosa* (*Pa*Pth) (PDB: 4FNO (4^th^ July 2012), PDB: 4FYJ (12^th^ December 2012), [24]) and a structure of the complex of *Ec*Pth with tRNA CCA-acceptor-TΨC domain [25] have also been reported. The comparison of amino acid sequences of these proteins indicate that the amino acid sequence identities of *Ab*Pth with these enzymes vary in the moderate range of 58% to 39% (Fig. S1 in File S1). A comparison of structural features of earlier known structures of *Ec*Pth, *Mt*pth and *Ms*Pth showed that considerable structural differences existed between them [4], [22], [23] which did not allow a global generalization. Therefore, it was considered to be essential to determine the structure of peptidyl-tRNA hydrolase from *Acinetobacter baumannii (Ab*Pth*)* for further enhancing the understanding of Pth structures as well as for the purpose of carrying out the structure based design of antibacterial compounds using peptidyl-tRNA hydrolase from *Acinetobactor baumannii* as the target protein. We report here the three-dimensional structures of unbound *Ab*Pth and two of its complexes with cytidine and uridine including binding studies using fluorescence spectroscopic and surface plasmon resonance studies. These structures revealed that cytidine and uridine bound to *Ab*Pth in the active site region and caused several conformational changes in the side chains of residues at the active site region in the protein resulting in the induced fits. The observed modes of binding of cytidine and uridine clearly provided a very useful information for the design of ligands as tight inhibitors of *Ab*Pth.

## Materials and Methods

### Cloning, Expression and Purification

The genomic DNA from *Acinetobacter baumannii* was isolated and Pth gene was amplified using forward and reverse primers (Pthf: 5- GGAATTC**CATATG**TCAAATATTTCGCTAATTG-3′) and (Pthr: 5- CCG**CTCGAG**TTAAGCTGGTTTATACGCATT-3′) respectively. The forward primer contained the site of recognition of *Nde1* (CATATG) and reverse primer contained the site of recognition of *xho1* (CTCGAG). The amplification of Pth gene was done with *Taq* DNA polymerase. A reaction was made using 5U of *Taq* DNA polymerase (MBI Fermentas, Vilnius, Lithuania) using PCR buffer containing 75 mM Tris-HCl, pH 8.8, 20 mM (NH_4_)_2_SO_4_, 0.01% Triton X-100, 1.5 mM MgCl_2_, 0.2 mM dNTPs and the genomic DNA of *Acinetobacter baumannii*. The amplified *Ab*Pth gene was used for ligation with linearized pGEM-T Easy vector. The ligation reaction was set up using the pGEM-T easy vector kit (Promega Corp, Wisconsin, USA). The reaction mix was incubated at 4°C overnight. The amount of amplified *Ab*Pth DNA required for ligation was calculated. The molar ratio of amplified *Ab*Pth gene and vector DNA was taken at a ratio of 5∶1. The pET vector DNA was completely digested with appropriate restriction enzymes (*Nde1* and *Xho1*). The digested plasmid was resolved on 0.8% agarose gel to remove the stuffer fragment. The plasmid band was purified by QIAEX-II gel extraction kit (Qiagen, Hilden, Germany) and stored at −20°C. The *Ab*Pth gene released from the recombinant pGEM-T Easy plasmid carrying complete open reading frame by digesting with *Nde1* and *Xho1* was then purified and ligated in the right reading frame (directional cloning) into already digested and purified pET-28a vector and transformed in *E. coli* DH5α cells. The recombinant clone was checked for the insert by colony PCR and also by restriction enzyme digestion with *Nde1* and *Xho1* in a 20 µl reaction containing 1.5 µl each of *Nde1* and *Xho1* and 2 µl NE Buffer (10X) and 15 µl of isolated plasmid (approximately 1 µg) which was incubated at 37°C for 4 h. In the pET prokaryotic system, the protein was expressed as an N-terminal fusion to 6 Histidine residues. The construct obtained with pET-28a was transformed in *E. coli* (Bl21 DE3) cells for expression of Pth. A single freshly transformed colony was inoculated in 10 ml LB containing 100 µg/ml kanamycin and was kept in water bath overnight at 37°C in shaking condition.

Overnight culture from primary inoculation (1%) was added in 1000 ml LB medium containing 100 µg/ml kanamycin and kept in water bath at 37°C in shaking condition till the optical density of the culture at 600 nm reached 0.4–0.6. 5 ml of this secondary culture was removed and kept at 4°C as an uninduced culture. The uninduced cells were used as the control. The remaining secondary culture of recombinant cells containing the inserts was induced with 1 mM IPTG at 37°C for 4 h. The induced and uninduced cells were harvested by centrifugation at 5000 rpm for 15 min, the supernatants were discarded and the pellet was stored at −70°C. The induction was checked on 10% sodium dodecyl sulfate-polyacrylamide gel electrophoresis (SDS-PAGE). The His-tagged protein containing 6 Histidine residues at the N-terminus was purified using Ni-NTA affinity resin chromatography.

The cell pellet (from two 500 ml induced bacterial cultures) was dissolved in 5 ml of Tris-HCl, pH 8.0 containing protease inhibitor cocktail (Roche, Basel, Switzerland). The sonication was done at 28 amplitude, 4 s pulse and 2 s rest was given. The cleared lysate was applied to a Ni-NTA Super-flow column pre-equilibrated in lysis buffer and purified using stepwise washing with 30 mM imidazole in lysis buffer followed by 300 mM imidazole in lysis buffer. The protein contents of each fraction were examined using 10% SDS-PAGE. The fractions corresponding to *Ab*Pth as indicated by its molecular mass were pooled. The imidazole was removed by dialysis in lysis buffer and the sample was concentrated using centricon with cut-off 3 kDa molecular mass (Millipore, MA, USA). The concentrated protein was further purified using Fast protein liquid chromatography (Bio-rad, CA, USA) using Superdex G50 column in buffer containing 20 mM Tris-HCl, 50 mM NaCl, 1.0 mM ethylenediaminetetraacetic acid (EDTA) and 5 mM of 2-mercaptoethanol (MPD), pH 8. The protein was concentrated using a centricon ultrafiltration of 3 kDa molecular mass cut off (Millipore, MA, USA). The purity of protein was established using SDS-PAGE which showed a single band (Fig. S2 in File S1).

### Binding Studies using Fluorescence Spectroscopy

The binding studies of *Ab*Pth with cytidine and uridine were carried out using fluorescence spectroscopic technique with spectrofluorometer, FP-6200 (Shimadzu, Kyoto, Japan). The fluorescence experiments were conducted by keeping the entrance and exit slit widths at 5 nm each and scanning it at the speed of 240 nm/min. *Ab*Pth was excited using an excitation wavelength of 280 nm. The fluorescence emission spectra of the protein were measured in the range of 300–550 nm at 298 K. The concentrations of ligands were varied as 5 µl, 10 µl, 15 µl, and 20 µl which were prepared from stock solutions of cytidine and uridine already prepared at a concentration of 1×10^−8^ mol/l. The protein concentration was kept constant at 1×10^−9^ mol/l. The spectral changes of *Ab*Pth with various concentrations were recorded and plotted. The least-squares fit of the fluorescence intensity changes for *Ab*Pth-cytidine and *Ab*Pth-uridine binding curves were obtained by Sigma Plot [26]. The error bars on the experimental points were estimated from the average of values which were obtained by repeating each experiment six times. The binding constants were calculated using the binding equation by Scatchard [27].

### Binding Studies using Surface Plasmon Resonance

Surface plasmon resonance (SPR) measurements were carried out for analyzing the bio-specific interactions using BIAcore T200 biosensor system (Biacore Inc., Uppsala, Sweden). The CM-5 research grade sensor chip, immobilization reagents (1-ethyl-3-(3-N,N-dimethylaminopropyl) carbodiimide (EDC), N-hydroxy succinimide (NHS), HBS-EP buffer and ethanolamine were procured from BIAcore Inc. All solutions were filtered using 0.22-µm membrane syringe filter and degassed before using. *Ab*Pth was covalently immobilized by a standard amine coupling procedure using the amine coupling kit supplied by the manufacturer. Throughout the immobilization experiment the flow rate of the running buffer was fixed at 10 µl/min with HBS-EP (pH 7.4, 0.01 M HEPES, 150 mM NaCl). The surface was activated using 70 µl of freshly mixed 100 mM NHS and 391 mM EDC for 7 min at 1∶1 (v/v) ratio. Upon activation, a 500 µg/ml solution of *Ab*Pth in 50 mM HEPES (pH 7.5) was injected for 8 min. The remaining active esters on the surface were quenched using 70 µl of 1.0 M ethanolamine (pH 8.5) for 7 min. Four different concentrations (0.8 µM, 1.5 µM, 2.5 µM and 4.5 µM) of each of the analytes, cytidine (S. D. Fine Chem. Ltd., Mumbai, India) and uridine (SISCO Research Laboratory, Mumbai, India) were passed over the immobilized *Ab*Pth at a flow rate of 10 µl/min. The regeneration of bound analytes was carried out by injecting 10 mM NaOH, pH 11.4 for 60 seconds, at a flow rate of 30 µl/min. These experiments were repeated six times and standard errors were calculated. The association rate constants, k_on_ (M^−1^s^−1^) and dissociation rate constants k_off_ (s^−1^) for the bindings and dissociations of cytidine and uridine to *Ab*Pth were obtained and the values of the equilibrium binding constants, K_D_ (M) were determined by the mass action relation, K_D_ = k_off_/k_on_ M using BIA evaluation 3.0 software as provided by the manufacturer.

### Crystallization

The purified samples of the unbound protein were crystallized using hanging drop vapor-diffusion method at 298 K by employing 24 well linbro crystallization plates. The initial crystallization screening experiments were carried out using Hampton research crystallization kit (HR2110 - HR2112). The protein was concentrated to 10 mgml^−1^ in 0.1 M HEPES buffer at pH 6.0. The diffractable crystals were obtained by equilibrating 10 µl protein drops (containing 7 µl protein solution and 3 µl reservoir solution) against a reservoir solution containing 25% (v/v) PEG-10000, 0.3 M MgCl_2_ and 0.1 M HEPES buffer at pH 6.0. Crystals grew to approximate dimensions of 0.4×0.4×0.2 mm^3^ in about 8 to 10 days. Fresh crystals were also obtained using a 1∶4 ratio of PEG-400 and PEG-1500 respectively in place of PEG-10000. The rest of the conditions were same. The crystals were soaked in the reservoir solutions containing 20 mg/ml cytidine and uridine in separate experiments. The crystals grown using 25% PEG-10000 were not stable in soaking conditions while those grown with PEG-400 and PEG-1500 were stable and were used for data collections on the two complexes. The soaking experiments were carried out for a minimum of 72 hours before mounting the crystals in the loops for data collections.

### X-ray Intensity Data Collections

Three X-ray intensity data sets were collected using 345 mm diameter MAR Research imaging plate scanner (Marresearch, Norderstedt, Germany) mounted on a rotating anode X-ray generator (Rigaku, Tokyo, Japan) operating at 50 kv and 100 mA. The crystals of unbound protein diffracted to 1.9 Å resolution while those of its complexes with cytidine and uridine diffracted to 1.87 Å and 1.40 Å respectively. The data were processed using program package HKL - 2000 [28]. The Matthews coefficients [29] were calculated to be 2.62 Å^3^/Da, 2.07 Å^3^/Da and 2.02 Å^3^/Da respectively. These corresponded to solvent contents of approximately 53.1%, 40.6% and 39.1% respectively.

### Structure Determination and Refinement of the Unbound *Ab*Pth

**Figure 1:**
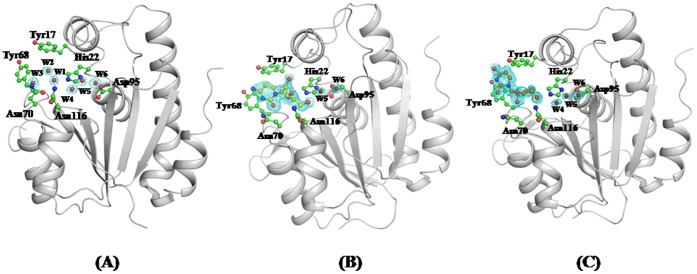
(Fo–Fc) difference electron density map calculated at 2.5 σ cut off before adding water oxygen atoms in the structure of unbound protein (A), cytidine in the structure of the complex with protein (B) and uridine in the structure of the complex with protein (C).

The structure of the unbound protein was determined with molecular replacement method using the coordinates of *Ec*Pth [4] as the search model. The coordinates obtained from structure determination were subjected to several cycles of refinement with REFMAC [30]. The whole protein chain was rebuilt segment by segment manually from the omit maps which were calculated by omitting the complete chain segment by segment. This was followed by several further cycles of refinement with REFMAC. The refined model was adjusted manually using programs coot [31] and O [32]. The structure model was improved by fitting the protein molecule in electron density maps calculated with (Fo–Fc) and (2Fo–Fc) coefficients. It was followed by nine rounds of refinement cycles. The model was improved by further manual model building. The additional cycles of refinement were carried out to locate water oxygen atoms. For the determination of positions of water oxygen atoms, the electron density peaks in the (Fo–Fc) map above 3σ were used. Six clear spherical electron density peaks were observed in the active site region. These were numbered as W1, W2, W3, W4, W5 and W6 (Fig. 1A). The water molecule W1 was of particular interest because it bridged two catalytically relevant residues Asn70 and His22. The B factors were refined isotropically. The refinement finally converged with overall values of 0.161 and 0.194 for the R_cryst_ and R_free_ factors. The C-terminal segment consisting of residues 181–193 which was reported to be flexible in the structures of peptidyl-tRNA hydrolases from other species was found to be well defined in the electron density of *Ab*Pth (Fig. S3 in File S1). The final structural validations carried out using PROCHECK [33] and MolProbity [34] showed 95.5% residues in the most favoured regions of the Ramachandran map [35] while Molprobity score was at 76 percentile. The atom by atom validation was also carried out using RSR [36] and correlation coefficient indicators [37]. The final refined coordinates were deposited in the PDB with an accession number 4JY7. The data processing and refinement parameters are listed in Table 1.

**Table 1:** Data collection and refinement statistics.

Crystallographic parameters	*Ab*PTH-Unbound	*Ab*PTH - Cytidine	*Ab*PTH -Uridine
PDB ID	4JY7	4JWK	4JX9
Space group	P21221	P212121	P212121
Unit-cell parameters ( 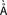 )			
a	34.5	34.2	33.9
b	58.2	64.4	65.8
c	109.3	78.6	75.7
Resolution range ( 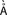 )	58.20–1.90	50.00–1.87	50.00–1.40
Highest resolution Shell ( 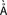 )	2.00–1.90	1.90–1.87	1.42–1.40
Number of observed reflections	143698	160559	366598
Number of unique reflections	18051	14968	34217
Vm Å^3^/Da	2.62	2.07	2.02
Solvent content (%)	53.1	40.6	39.1
I/σ (I)	16.5 (4.2)	37.3 (4.4)	61.1 (10.1)
Completeness (%)	99.8 (99.6)	100 (100)	99.9 (100)
R_sym_ (%)	10.0 (53.7)	8.4 (53.7)	4.2(23.0)
**Refinement**			
R_cryst_ (%)	16.1	18.0	19.2
R_free_ (%)	19.4	22.1	20.9
Protein atoms	1476	1476	1476
Ligand atoms	–	17(1)	17 (1)
Water oxygen atoms	200	136	216
Atoms of glycerol molecules (3)	18	–	–
Atoms of 1,2-ethanediols (2)	8	–	–
Atoms of diethylene glycols (2)	14	–	–
Atoms of acetate ion (1)	4	–	–
**Ramachandran plot statistics**			
Residues in the preferred regions (%)	95.5	99.5	97.9
Residues in the allowed regions (%)	3.9	–	1.6
Residues in the disallowed region (%)	0.6 (Tyr 68)	0.5 (Tyr 68)	0.5 (Tyr 68)
***R. m. s. deviations from ideal values***			
Bond lengths ( 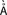 )	0.017	0.005	0.004
Bond angles (^o^)	1.8	1.1	1.1
Dihedral angles (^o^)	15.5	16.2	13.9
**Mean B factor ( 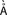 ^2^)**			
Main chain atoms	19.5	26.1	11.1
Side chains	26.8	28.3	11.9
Ligands	–	(Cytidine)	(Uridine)
Water atoms	37.2	40.6	25.3
For all atoms	23.5	28.5	13.5

Values in parentheses are for the highest resolution shell.

### Determinations and Refinements of Structures of the Complexes of *Ab*Pth with Cytidine and Uridine

The structures of the complexes of *Ab*Pth with cytidine and uridine were determined with molecular replacement method using coordinates of the structure of unbound *Ab*Pth (PDB code: 4IKO) as the search model. Since the space group of the crystals of the unbound protein was different from those of the complexes with cytidine and uridine, the use of molecular replacement method was essential for obtaining the solution of the structures of the complexes. Several cycles of refinement calculations carried out with REFMAC [30] were followed with manual model buildings using programs coot [31] and O [32]. When the refinements reached the stage with R_cryst_ factors of approximate value of 0.25, the Fourier maps with (Fo–Fc) and (2Fo–Fc) coefficients were calculated for both structures. The extra non-protein electron densities were observed in the active site region into which molecules of cytidine (Fig. 1B) and uridine (Fig. 1C) were modeled and refined. As a result of the bindings of cytidine and uridine at the active site, the densities for the side chains of Asn70, Asp98 and Asn116 were observed at positions which were different from their positions in the unbound structure of *Ab*Pth (Figs. S4A and S4B in File S1) indicating a large shift for accommodating the ligands. The coordinates of cytidine and uridine were included in the further rounds of refinement calculations. The positions of cytidine and uridine were adjusted manually. At this stage, fresh difference Fourier (Fo–Fc) maps were calculated. The positions of 136 and 216 water oxygen atoms were determined for the structures of the complexes of *Ab*Pth with cytidine and uridine respectively. These were also included in the subsequent cycles of refinements. After five more rounds of refinements for both structures, the values of R_cryst_/R_free_ factors converged to 0.180/0.221 and 0.192/0.209 for the structures of the complexes with cytidine and uridine respectively. The final structural validations carried out using PROCHECK [33] and Mol Probity [34] showed 99.5% and 97.9% of residues in the most favoured regions of the Ramachandran map [35] for the complexes with cytidine and uridine respectively. The values of RSR/correlation coefficients for cytidine and uridine were found to be were 38%/68% and 42%/70% respectively. The coordinates of these two structures have been submitted to the protein data bank (PDB) with accession codes of 4JWK and 4JX9. The details of data processings and refinements have been included in Table 1.

## Results

### Analysis of Binding using Fluorescence Spectroscopic Techniques

**Figure 2:**
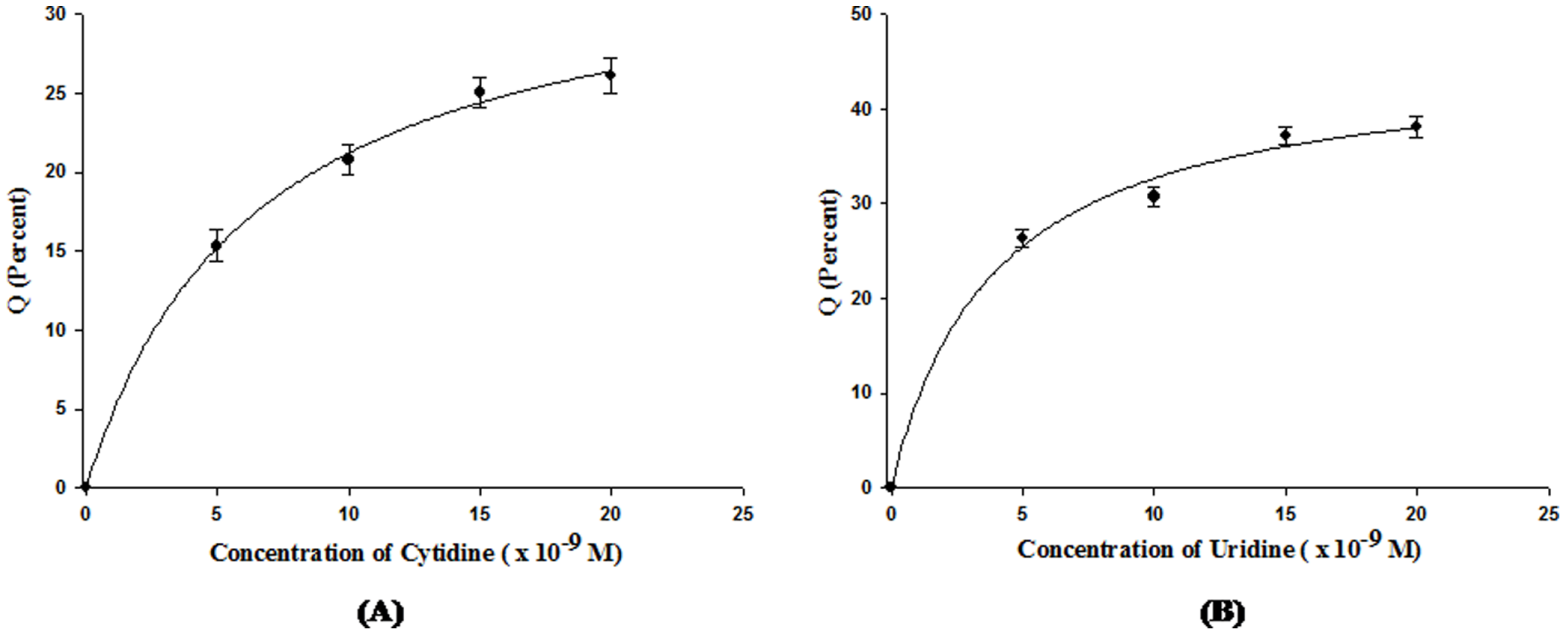
Binding curves (A) for the binding of cytidine to *Ab*Pth and (B) for the binding of uridine to *Ab*Pth showing the changes in fluorescence intensities (ΔF/Fo) at 347 nm as the ligands to protein ratios were increased (5 µl, 10 µl, 15 µl and 20 µl). The errors on the experimental points are indicated.

The binding studies of *Ab*Pth with cytidine and uridine were carried out using fluorescence spectroscopic techniques. The concentration of protein was kept fixed while the concentrations of ligands were increased and the bindings of both compounds were analyzed. It was observed that the bindings of both cytidine and uridine quenched the intrinsic fluorescence intensity of the protein at 347 nm which indicated that the compounds bound to *Ab*Pth. The observed fluorescence data were used for obtaining the fluorescence quenching coefficient: Q = (Fo−F)/Fo, where F is the measured fluorescence and Fo is the fluorescence in the absence of ligands. The values of Q were plotted in percentage against the different concentrations of ligands. The R^2^ values, which provide an index of the goodness of fit of the curves were obtained using Sigma Plot 8.0 [26] (Fig. 2) and were determined as 0.98 for cytidine, and 0.99 for uridine. The binding constants were calculated using the binding equation described by Scatchard [27]. The approximate values of binding constants were found to be 4.79±0.21×10^−9^ M and 2.11±0.32 × 10^−9^ M respectively indicating that both compounds bound to *Ab*Pth with high affinities in the range of nanomolars.

### Surface Plasmon Resonance

**Figure 3:**
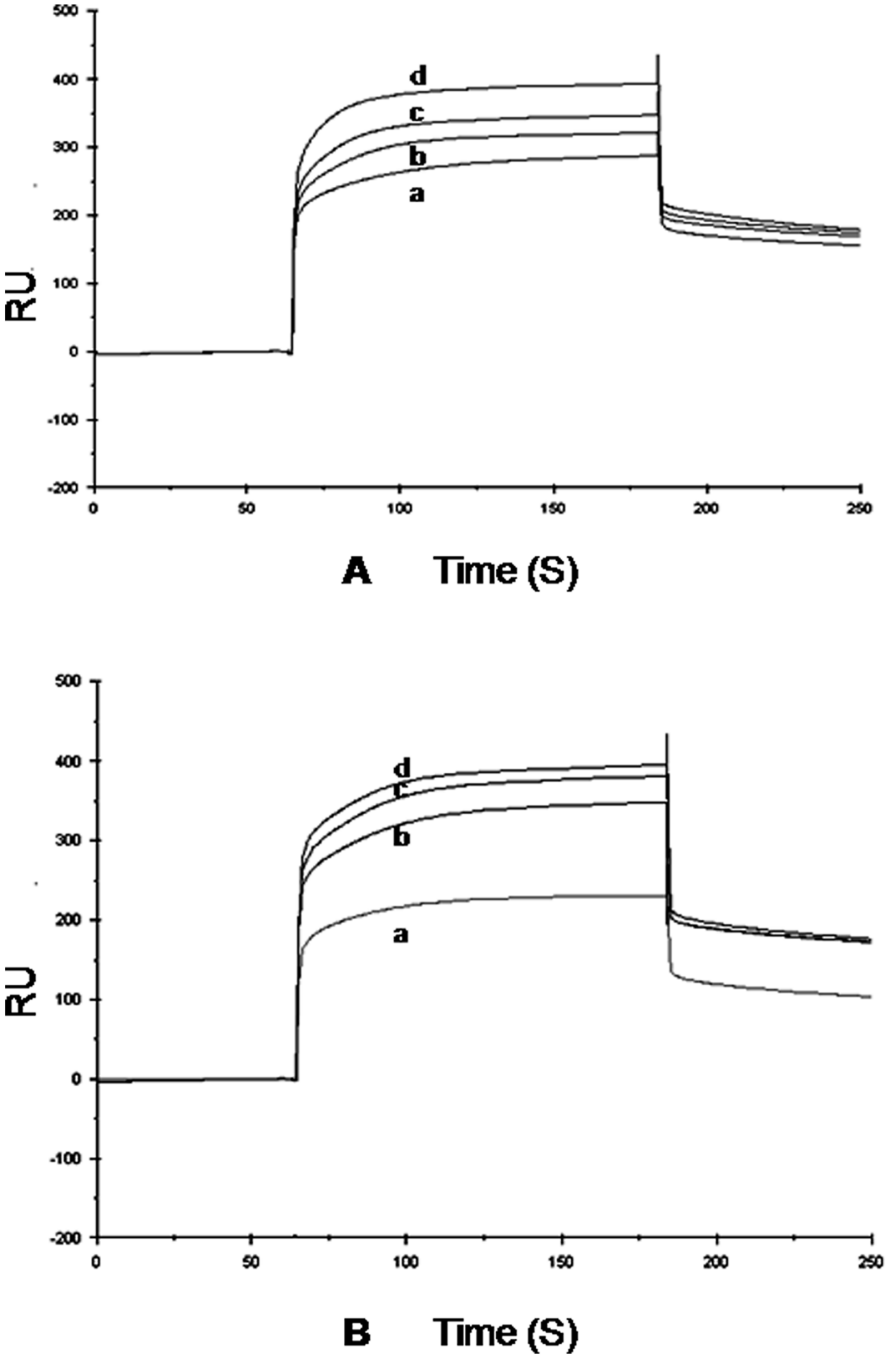
The SPR sensograms for the bindings of (A) cytidine and (B) uridine to protein, *Ab*Pth. The protein was immobilized on CM-5 chip and the increasing concentrations (a) 0.8 µM, (b) 1.5 µM, (c) 2.5 µM and (d) 4.5 µM of analytes cytidine and uridine were used in mobile phase in separate experiments corresponding to curves in (A) and (B) respectively.

The binding studies of *Ab*Pth with uridine and cytidine were also carried out using real time SPR spectroscopy. The SPR sensograms illustrating the nature of association and dissociation curves for cytidine and uridine with immobilized *Ab*Pth are given in Fig. 3. The increase in resonance units (RUs) from the baseline shows the binding of ligand to the immobilized protein. The plateau line indicates a steady state equilibrium phase of interactions between protein and the ligand while the reduction in the values in RU from the plateau shows the dissociation phase. As seen from Fig. 3A and 3B, the dissociation phase is slightly slower in the case of uridine than that of cytidine. This showed that uridine has a slightly higher affinity than that of cytidine. The overall fitting of the observed data to a Langmuir 1∶1 association model obtained with BIA-evaluation 3.0 software package provided the values of 5.3±0.4 × 10^−9 ^M and 1.4±0.2 × 10^−9 ^M for the equilibrium binding constants (K_Ds_) for cytidine and uridine respectively.

### Overall Structure of the Unbound *Ab*Pth

**Figure 4:**
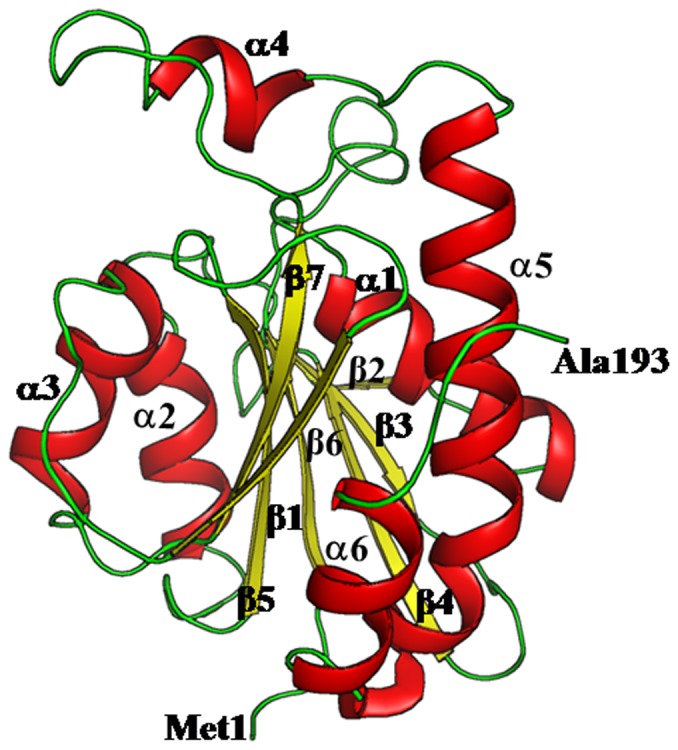
Showing overall folding of the polypeptide chain of *Ab*Pth. The secondary structure elements are indicated where α-helices, α1 (residues: 24–36), α2 (residues: 72–82), α3 (residues: 116–125), α4 (residues: 146–151), α5 (residues: 156–176) and α6 (residues: 180–188) are shown in red and β-strands, β1 (residues: 5–10), β2 (residues: 41–43), β3 (residues: 48–52), β4 (residues: 58–64), β5 (residues: 89–96), β6 (residues: 103–108) and β7 (residues: 130–136) are shown in green. The N-terminal and C-terminal residues are also indicated.

**Figure 5:**
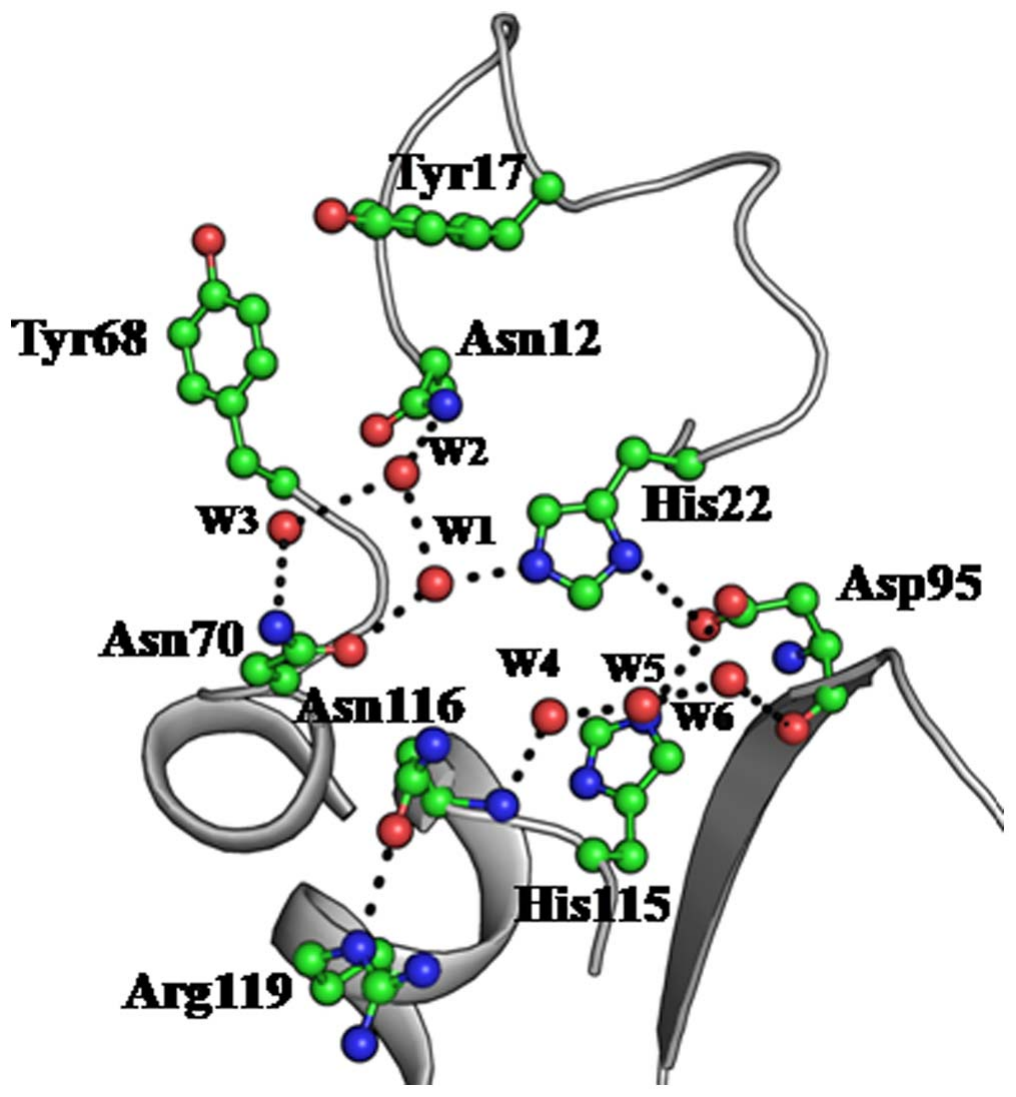
The hydrogen bonded network between protein atoms and water molecules as well as between water molecules in the active site region.

The refined model of *Ab*Pth consists of 1476 protein atoms from 193 amino acid residues, 200 water oxygen atoms, 18 atoms from three glycerol molecules, two molecules of 1, 2-ethanediol, two molecules of diethyleneglycol and one acetate ion. The polypeptide chain of *Ab*Pth adopted an α/β fold with seven β-strands, β1 (Ser5 - Leu10), β2 (Lys41 - Asp43), β3 (Gly48 - Arg52), β4 (His58 - Leu64), β5 (Ala89 - Glu96), β6 (Val103 - Thr108) and β7 (His130 - Ile136) and six α-helices, α1 (Ala24 - Tyr36), α2 (Ser72 - Phe82), α3 (Leu116 - Ile125), α4 (Ser146 - Gly151), α5 (Asn156 - Gln179) and α6 (Pro181 - Asn188) (Fig. 4). A twisted β-sheet is formed in the centre of the molecule which is surrounded by seven α-helices. The active site is located in a cleft which is formed with segments consisting of amino acid residues, Leu10 - Ala24, Pro65 - Gly72, Leu90 - Pro101 and Pro139 - Leu150. The residues related to the catalytic action are His22, Asn70, Asp95 and Asn116 [23]. In the structure of unbound protein, six water molecules, W1, W2, W3, W4, W5 and W6 have been observed in the active site region (Fig. 5). W1 is located between Asn70 and His22 and forms hydrogen bonds with both residues while W2 and W3 form a bridge between Asn12 and Asn70. W1 and W2 are linked through a hydrogen bond. W4, W5 and W6 are linked through hydrogen bonds in a linear chain and interconnect Asp95 and Asn116. On the other hand Asn116 is hydrogen bonded to Arg119.

**Figure 6:**
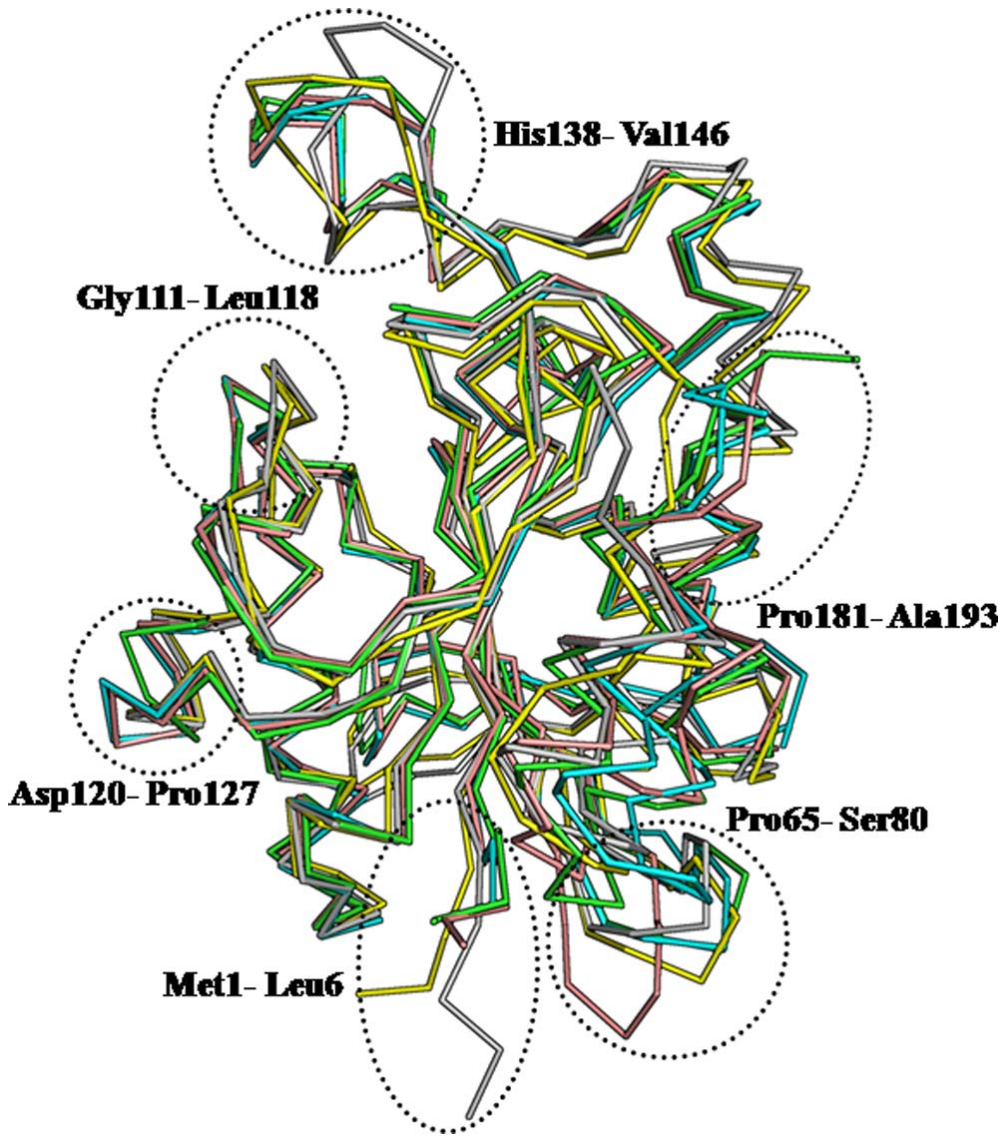
The superimpositions of the C^α^ traces of *Ab*Pth (green), *Ec*Pth (cyan), *Pa*Pth (pink), *Mt*Pth (yellow) and *Ms*Pth (grey). The flexible regions consisting of residues, (Met1 - Leu6), (Pro65 - Ala80), (Gly111 - Leu118), (Asp120 - Pro127), (His138 - Val146) and (Pro181 - Ala193) are marked by dotted circles.

The overall structure of *Ab*Pth is similar to that of *Pa*Pth (PDB: 4FNO), *Ec*Pth [4], *Mt*Pth [23] and *Ms*Pth [22] with r.m.s. shifts for the C^α^ traces being 1.0 Å, 1.0 Å, 1.5 Å and 1.7 Å respectively. As seen from Fig. 6, the paths of the polypeptide chains of *Ab*Pth (green), *Pa*Pth (pink), *Ec*Pth (cyan), *Mt*Pth (yellow) and *Ms*Pth (grey) differ greatly in several regions including segments with residues: Met1 - Leu6, Pro65 - Ser80, Gly111 - Leu118, Asp120 - Pro127, His138 - Val149 and Pro181 - Ala193. These structural variations in Pth enzymes from different species (PDB: 4FNO, PDB: 4IKO, [4], [22], [23]) were also reflected in terms of certain distances which were between the pairs of atoms. The distances between Asp98 O^δ2^ on one side of the wall of active site region and Gly113 C^α^ from the wall on the opposite side (*Ab*Pth numbering scheme) were found to be 6.1 Å, 6.8 Å, 6.6 Å, 3.3 Å and 4.5 Å in *Ab*Pth, *Pa*Pth, *Ec*Pth, *Mt*Pth and *Ms*Pth respectively. Another set of distances measured between Gly113 C^α^ and Ser146 C^α^, also from opposite sides were found to be 15.3 Å, 15.0 Å, 14.9 Å, 11.6 Å and 11.3 Å in *Ab*Pth, *Pa*Pth, *Ec*Pth, *Mt*Pth and *Ms*Pth respectively. The distance between Asn70 O^δ1^ and Asp98 O^δ2^ is found to be 15.7 Å in the unbound *Ab*Pth. The corresponding distances in *Pa*Pth, *Ec*Pth, *Mt*Pth and *Ms*Pth were 20.9 Å, 16.9 Å, 16.2 Å and 17.2 Å respectively. The lengths of Pth molecules as measured in one of the directions in terms of distances between Tyr83 C^α^ and Gly140 C^α^ were found to be 41.0 Å, 41.1 Å, 40.0 Å, 42.1 Å and 43.1 Å for *Ab*Pth, *Pa*Pth, *Ec*Pth, *Mt*Pth and *Ms*Pth respectively. These values indicated that *Ab*Pth, *Ec*Pth and *Pa*Pth are slightly more compact molecules but possessed wider opening of the gate to the active site cleft than those of *Mt*Pth and *Ms*Pth. The minimum distance between the side chains of two tyrosine residues, Tyr17 and Tyr68 which are believed to be involved in the stacking with RNA bases is 4.1 Å in *Ab*Pth. The corresponding distances in *Pa*Pth, *Ec*Pth, *Mt*Pth and *Ms*Pth are 3.7 Å, 3.8 Å, 6.8 Å and 5.7 Å respectively. In *Ab*Pth, Tyr68 adopted a conformation with φ, ψ values of 76.6°, 148.3° which falls in the disallowed region of Ramachandran map [35]. The conformation of the corresponding residue, Phe66 in *Ec*Pth with φ, ψ values of 92.5°, 147.5° was also observed in the disallowed region [4] but the same residue when *Ec*Pth formed a complex with CCA-acceptor-TΨC domain of tRNA moiety adopted a conformation with φ, ψ values of −60.1°, 157.3° which belonged to the fully allowed region. In this regard, it may be mentioned that the structure of the so-called unbound state in *Ec*Pth contained the C-terminal tripeptide of a symmetry related neighboring molecule at the active site cleft [4] while in the complex with tRNA moiety, the active site cleft was empty [25]. It indicated that Phe66 in *Ec*Pth adopted a conformation in the disallowed region with external chemical moiety in the active site region and it adopted the conformation in the allowed region when empty. The φ, ψ values for the corresponding residues in *Pa*Pth [4FNO], *Mt*Pth [22] and *Ms*Pth [23] were −73.0°, 167.7°, −57.0°, 150.1° and −81.6°, 149.1° respectively which were in the fully allowed regions of the Ramachandran map.

There is another segment, Asp120 - Pro127 that showed differences in the conformations in Pth from various species. This segment includes a part of α-helix, α3 (residues, 116–125). The values of φ, ψ (−85.4°, −2.8°; −114°, −49.5°; −47.9°, −38.8°; −62.5°, −25.3°; −107.7°, −30.4°; −112.0°, −5.2°; 85.7°, 179.1°; −93.6°, −4.7°) for the residues in this segment of *Ab*Pth indicated a considerable deviation from the normal values for α-helical conformations. This may be due to the presence of Pro123 in the middle of the segment. The corresponding residues in Pth from other species are either Ala or Ser (Fig. S1 in File S1). The non-ideal conformation of helix α3 in *Ab*Pth may be more favourable for the binding of substrates to the enzyme. The corresponding residues adopt normal α-helical conformations in Pth of other species. Furthermore, the C-terminal segment (residues, 181–193) in *Ab*Pth which also includes α-helix, α6 (residues, 181–188) was found to be very well defined in the electron density (Fig. S3 in File S1) unlike those of other Pth structures ([4], [22], [23], PDB: 4FNO). It may also be mentioned here that the conformation of the N-terminal hexapeptide has not been in general defined well in the previously determined structures of Pth enzymes. It was reported in the case of native where the last three residues from the neighboring molecule were involved in the intermolecular interactions and these residues were slightly buried in the active site cleft of the molecule.

### Structure of the Complex of *Ab*Pth with Cytidine

**Figure 7:**
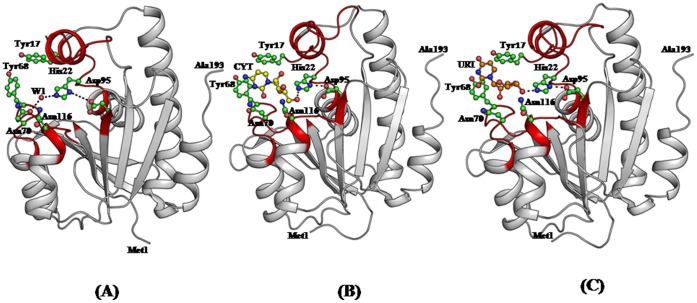
Showing structures of (A) native *Ab*Pth with a hydrogen bonded water molecule W1 with both His22 and Asn70. (B) *Ab*Pth with bound cytidine and (C) *Ab*Pth with uridine. The dotted lines indicate hydrogen bonds.

The structure of the unbound *Ab*Pth showed the presence of six water molecules in the active site region of which W1 is hydrogen bonded to both Asn70 and His22 (Fig. 7A). However, in the structure of the complex with cytidine (Fig. 7B) as a result of binding of cytidine to protein four water molecules, W1, W2, W3 and W4 were expelled from the structure. Furthermore, the binding of cytidine in the active site caused drastic conformational changes in the side chains of Asn70, Asp98 and Asn116. These side chains in the complex moved away, from their original positions which were observed in the unbound structure, for accommodating ligand molecule. These structural changes were also reflected in terms of distances between Gly113 C^α^ and Asp98 O^δ2^ and Asn70 O^δ1^ and Asp98 O^δ2^ which were found to increase from 6.1 Å to 8.2 Å, 15.7 Å to 22.7 Å respectively indicating that the segments containing Gly113, Asp98 and Asn70 moved away to make space for the ligand. The complete reversals of the side chains of Asn70 and Asp98 are noteworthy. After having introduced all these changes, cytidine molecule got well stabilized in the cleft with the help of atleast seven hydrogen bonds and 72 van der Waals contacts (distances upto 4.2 Å). The pyrimidine ring of cytidine molecule is seen well stacked with the side chain of Tyr68 which was found to adopt a conformation in the disallowed region with φ, ψ values of 74.9°, 148.3°. This conformation is similar to that observed in the unbound state. It appears that the observed disallowed conformation of Tyr68 is favorable to generate good the stacking interactions with RNA bases.

### Structure of the Complex of *Ab*Pth with Uridine

The structure of the complex of *Ab*Pth with uridine is shown in Fig. 7C. Since uridine occupied the space in active site cleft, it displaced three water molecules, W1, W2 and W3 while the position of W4 was altered. It also caused large conformational changes in Asn70, Arg71, Asp98 and Asn116. In this case, distance between Gly113 C^α^ and Asp98 O^δ2^ and between Asn70 O^δ1^ and Asp98 O^δ2^ increased from 6.1 Å to 7.8 Å and 15.7 Å to 22.7 Å respectively as the segments containing residues Gly113 and Asp98 as well as the side chains of Asn70 and Asp98 move away to accommodate the ligands. The binding of uridine was stabilized with the help of seven hydrogen bonds and more than 100 van der Waals contacts (distances upto 4.2 Å). As observed in the structure of the complex of *Ab*Pth with cytidine, the pyrimidine ring of uridine is also well stacked with the aromatic side chain of Tyr68. The conformation of Tyr68 with φ, ψ values of 75.4°, 148.3° is similar to that observed in the structure of unbound *Ab*Pth and its complexes with cytidine.

## Discussion

The equilibrium constants (K_Ds_) for the binding of cytidine and uridine to *Ab*Pth using fluorescence spectroscopic technique were found to be 4.79×10^−9^ M and 3.11×10^−9^ M respectively. Similar values of 5.3×10^−9 ^M and 2.4×10^−9^ M for the equilibrium constants (K_Ds_) for the binding of cytidine and uridine respectively were obtained using SPR techniques. The high values of binding constants showed that the two compounds bound to *Ab*Pth firmly. The buried surface areas as estimated from the structures of the complexes of *Ab*Pth with cytidine and uridine were estimated to be 202 Å^2^ and 198 Å^2^ respectively [38]. These values when compared with their molecular weights of 243 Da and 244 Da respectively indicated that the buried areas were of the comparable order as observed in other established complexes [39]–[45]. Thus it confirmed that cytidine and uridine formed stable complexes with *Ab*Pth.

**Figure 8:**
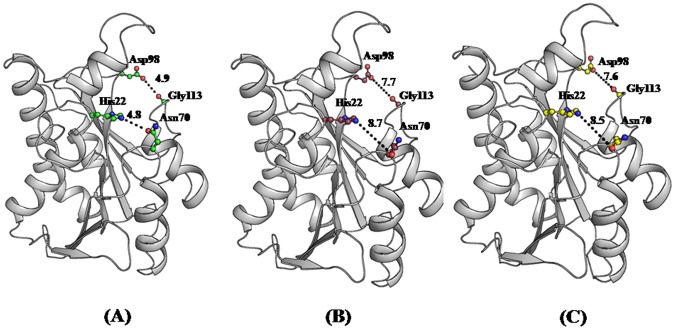
The distances between Asp98 O^δ2^ and Gly113 O and His22N^ε2^ and Asn70 O^δ1^ in the structure of the (A) unbound protein and in its complexes with (B) cytidine and (C) uridine.

The observed conformational changes in the protein upon binding to cytidine and uridine indicated a huge capability of this protein to recognize various peptidyl-tRNA molecules with peptide moieties having different amino acid sequences through an induced fit approach. In the structure of unbound *Ab*Pth the minimum distance between the atoms on the opposite sides of the entry point (Asp98 O^δ2^– Gly113 O) was 4.9 Å while the shortest separation between the opposite walls at the active site (His22 N^ε2^– Asn70 O^δ1^) was 4.8 Å (Fig. 8A). The corresponding distances in the complex with cytidine were 7.7 Å and 8.7 Å respectively (Fig. 8B) whereas these distances in the complex with uridine were 7.6 Å and 8.5 Å respectively (Fig. 8C). The minimum distance as measured between Gly113 C^α^ O atom on one side to Asp98 O^δ2^ atom on the opposite side of the entrance gate increased from 4.9 Å to 7.7 Å and 7.6 Å to allow the entry of cytidine and uridine respectively into the active site. After having entered in the active site region, the binding of cytidine caused expulsions of four out of six water molecules from the active site cleft while uridine replaced three out of six water molecules and shifted the position of fourth. In the unbound *Ab*Pth, the distances between Asn70 O^δ1^ and His22 N^ε2^ was 4.8 Å which changed to 8.7 Å on the binding to cytidine while upon binding to uridine it changed to 8.5 Å. These observations clearly indicated an induced fit mode of inhibitor binding in *Ab*Pth.

**Figure 9:**
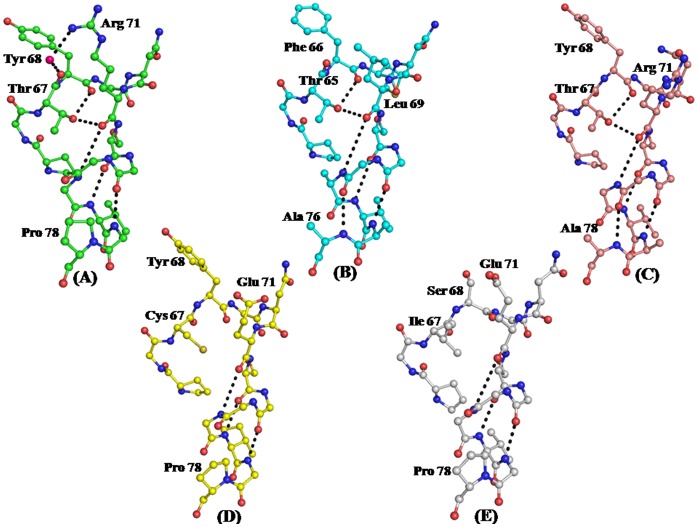
Structural environment of the segment Pro 65 - Pro 78 where the conformation of Tyr68 is observed in (A) disallowed region in *Ab*Pth (b) disallowed region in *Ec*Pth while the corresponding residues were found with allowed conformations in (C) *Pa*Pth, (D) *Mt*Pth and (E) *Ms*Pth.

On the other hand an identical yet disallowed conformation of Tyr68 in both unbound and bound states of *Ab*Pth was observed. The examination of the structures of the complexes of *Ab*Pth with cytidine and uridine clearly showed favourable stacking interactions involving Tyr68 and aromatic rings of RNA bases. Therefore, the disallowed conformation of Tyr68 as supported by the structure of *Ab*Pth is of functional significance. This is in huge contrast to those observed in Pth structures from other species ([4], [22], [23], PDB: 4FNO). As seen from Fig. 9A, the observed conformation of Tyr68 in *Ab*Pth is locked with extensive interactions from neighboring residues. In contrast, in the structure of *Ec*Pth, a so-called native state structure has actually a bound C-terminal tripeptide from a neighboring symmetry related protein molecule that blocked the entry to the active site and hence in reality it represents a complexed state. On the other hand, another structure of *Ec*Pth with a truncated RNA molecule which is actually a complex but its active site is empty and hence appears to be an unbound state as far as the binding through active site is concerned. It is noteworthy that in the case of former, Phe66 (the residue corresponding to Tyr68 in *Ab*Pth adopted a conformation in the disallowed region while in the latter case it preferred to be in the allowed region. As seen from Fig. 9B, the conformation of residue Phe66 in *Ec*Pth is poorly stabilized as the residues corresponding to Met66, Arg71, and Pro78 of *Ab*Pth, are Thr64, Leu69 and Ala76 which do not provide stabilizing interactions of *Ab*Pth (Figs. 9A and 9B). Similar networks of interactions in the segment containing Tyr68 were lacking in peptidyl-tRNA hydrolases from other species (Fig. 9C–E). These data showed that *Ab*Pth is better designed for carrying out a more efficient enzymatic action on peptidyl-tRNAs.

### Conclusions

**Figure 10:**
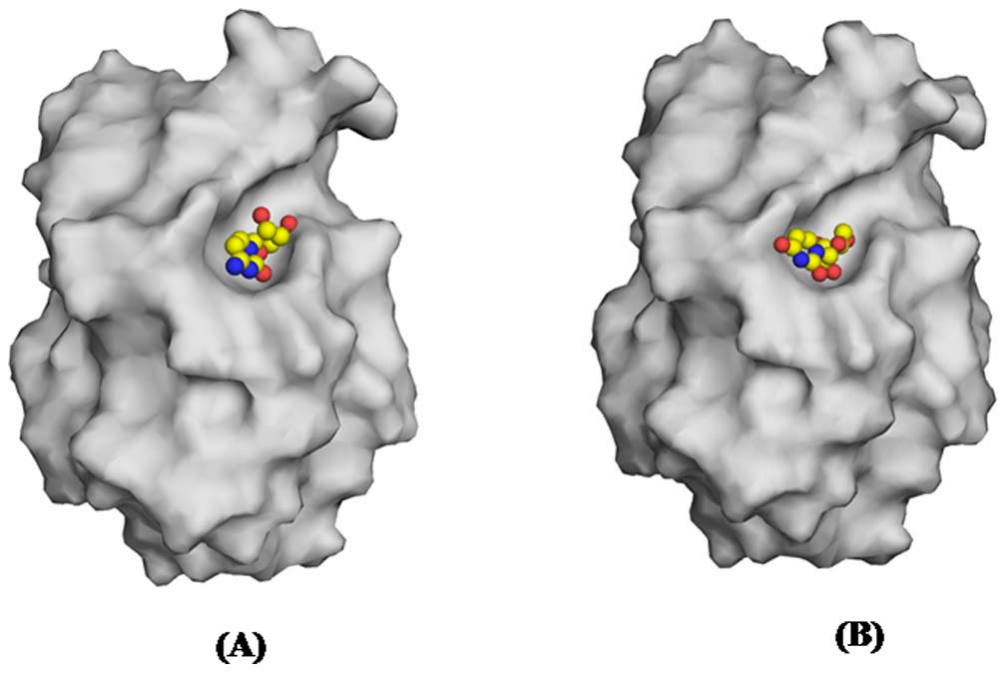
Grasp representation of the fitting of (A) cytidine and (B) uridine in the active site pocket of *Ab*Pth.

Three important goals for carrying out structural studies of peptidyl-tRNA hydrolase (Pth) were (i) to determine structural features of Pth including the stereochemical details of the active site, (ii) to explain the mechanism of catalytic action and (iii) to understand the mode of ligand binding for the design of inhibitors. The goals (i) and (ii) had been partially understood from previous studies while the information on goal (iii) was completely missing. The present studies have provided a clear understanding of the mode of inhibitor binding in peptidyl-tRNA hydrolases. The structure determinations of the complexes of *Ab*Pth with cytidine and uridine have revealed the nature of interactions between the protein atoms and bound compounds. It revealed the identity of amino acid residues that are involved in the interactions and are important for recognition. It has further shown that as a result of interactions of cytidine and uridine with protein atoms, a widening of the entrance gate to the active site region has occurred (Fig. 8A–C). Furthermore, when the ligands, cytidine and uridine entered into the active site region through the widened gate, large scale conformational changes were induced to attain the tight fittings of the binding compounds. It was necessary to widen the space at the active site region for accommodating the ligands. The induced fit of binding as observed here suggests that *Ab*Pth is capable of binding to a variety of peptide sequences of different peptidyl-tRNAs. These studies also showed that the conformation of Tyr 68 in *Ab*Pth as observed in the disallowed region was important for the stacking of RNA bases. Since the conformations of the corresponding residues in Pth from other species were less favorable for stacking or required induction on binding, these were less efficient enzymes than *Ab*Pth. All these are important and useful observations for the structure based ligand design. These results also highlighted that even though the usual substrate, peptidyl-tRNA is a large molecule, the inhibitors can be small compounds that block the active site cleft as observed in the complexes of cytidine (Fig. 10A) and uridine (Fig. 10B). Therefore, the design of compounds should include a moiety that stacks with Tyr68, it is able to expel water molecules from the active site region and forms hydrogen bonded interactions with His22, Asn70 and Asn116 while it should form van der Waals contacts with residues Asn12, Tyr17, His22, Tyr68, Met69, Asn70, Asn116 and Arg119. Using these detailed structural information, a rational structural-based drug design is achievable and the tight inhibitors of peptidyl-tRNA hydrolase can be prepared. The observed induced fit of binding as observed here here suggests that *Ab*Pth has a capability of binding to a variety of peptide sequences of different peptidyl-tRNAs.

## Supporting Information

File S1 Figure S1. Sequence alignments of peptidyl tRNA hydrolases (Pths) from *Acinetobacter baumannii* (*Ab*Pth) with *Pseudomonas aeruginosa* (*Pa*Pth) *Escherichia coli* (*Ec*Pth), *Mycobacterium tuberculosis* (*Mt*Pth) and *Mycobacterium smegmatis* (*Ms*Pth) whose crystal structures are known. The overall sequence identities vary from 58% to 39%. The flexible regions consisting of residues, Met1 - Leu6, Pro65 - Ser80, Gly111 - Leu118, Ile121 - Pro127, Pro139 - His148 and Pro181 - Ala193 are highlighted in grey. The probable residues involved in the catalysis are highlighted in yellow. The secondary structure elements α-helices (cylinders) and β-strands (arrows) are also indicated above the sequences. Figure S2. Showing results of polyacrylamide gel electrophoresis for *Ab*Pth. Lane A: molecular weight markers, 116.0 kDa - β-galactosidase, 66.2 kDa - bovine serum albumin, 45.0 kDa - ovalbumin, 35.0 kDa - lactate dehydrogenase, 25.0 kDa - ribonuclease, 18.4 kDa - β-lactoglobulin and 14.4 kDa - lysozyme; Lane B: purified protein, *Ab*Pth. Figure S3. Showing electron densities for the C-terminal segment, Pro181– Ala193 (A) |Fo–Fc| map at 2.5 σ cut off and (B) |2Fo–Fc| map at 1 σ cut off. Figure S4. Bindings of cytidine (A) and uridine (B) showing large conformational changes with side chains of Asn70, Asp98 and Asn116. The electron densities from the initial (Fo–Fc) map at 2.5 σ cut off.
(DOC)
