# Supplementary material for: The Mode of Inhibitor Binding to Peptidyl-tRNA Hydrolase: Binding Studies and Structure Determination of Unbound and Bound Peptidyl-tRNA Hydrolase from Acinetobacter baumannii
Source: PLoS One. 2013 Jul 3;8(7):e67547. doi: 10.1371/journal.pone.0067547 (PMC3701073; doi:10.1371/journal.pone.0067547)
Supplement: File S1 — Figure S1. Sequence alignments of peptidyl tRNA hydrolases (Pths) from Acinetobacter baumannii (AbPth) with Pseudomonas aeruginosa (PaPth) Escherichia coli (EcPth), Mycobacterium tuberculosis (MtPth) and Mycobacterium smegmatis (MsPth) whose crystal structures are known. The overall sequence identities vary from 58% to 39%. The flexible regions consisting of residues, Met1 - Leu6, Pro65 - Ser80, Gly111 - Leu118, Ile121 - Pro127, Pro139 - His148 and Pro181 - Ala193 are highlighted in grey. The probable residues involved in the catalysis are highlighted in yellow. The secondary structure elements α-helices (cylinders) and β-strands (arrows) are also indicated above the sequences. Figure S2. Showing results of polyacrylamide gel electrophoresis for AbPth. Lane A: molecular weight markers, 116.0 kDa - β-galactosidase, 66.2 kDa - bovine serum albumin, 45.0 kDa - ovalbumin, 35.0 kDa - lactate dehydrogenase, 25.0 kDa - ribonuclease, 18.4 kDa - β-lactoglobulin and 14.4 kDa - lysozyme; Lane B: purified protein, AbPth. Figure S3. Showing electron densities for the C-terminal segment, Pro181– Ala193 (A) |Fo–Fc| map at 2.5 σ cut off and (B) |2Fo–Fc| map at 1 σ cut off. Figure S4. Bindings of cytidine (A) and uridine (B) showing large conformational changes with side chains of Asn70, Asp98 and Asn116. The electron densities from the initial (Fo–Fc) map at 2.5 σ cut off. (DOC) [file pone.0067547.s001.doc]

**The mode of inhibitor binding to peptidyl-tRNA hydrolase: Binding studies and structure determination of unbound and bound peptidyl-tRNA hydrolase from *Acinetobacter Baumannii***

Sanket Kaushik^1^, Nagendra Singh^1^, Shavait Yamini^1^, Avinash Singh^1^, Mau Sinha^1^, Ashish Arora^2^, Punit Kaur^1^, Sujata Sharma^1^, Tej P. Singh^1*^

^1^Department of Biophysics, All India Institute of Medical Sciences, New Delhi, India

^2^Central Drug Research Institute, Lucknow, India

**Keywords:** Structure, Peptidyl**-**tRNA hydrolase, Enzyme, *Acinetobacter baumannii*, Inhibitor, Induced conformation.

^*^**Author for correspondence:**

T. P. Singh

Department of Biophysics

All India Institute of Medical Sciences

Ansari Nagar, New Delhi - 110 029

India

Tel : +91-11-2658-8931

FAX: +91-11-2658-8663

e-mail: [tpsingh.aiims@gmail.com](mailto:tpsingh.aiims@gmail.com)

**LEGENDS TO FIGURES**

**FIGURE S1** Sequence alignments of peptidyl tRNA hydrolases (Pths) from *Acinetobacter baumannii* (*Ab*Pth) with *Pseudomonas aeruginosa* (*Pa*Pth) *Escherichia coli* (*Ec*Pth), *Mycobacterium* *tuberculosis* (*Mt*Pth) and *Mycobacterium smegmatis* (*Ms*Pth) whose crystal structures are known. The overall sequence identities vary from 58% to 39%. The flexible regions consisting of residues, Met1 - Leu6, Pro65 - Ser80, Gly111 - Leu118, Ile121 - Pro127, Pro139 - His148 and Pro181 - Ala193 are highlighted in grey. The probable residues involved in the catalysis are highlighted in yellow. The secondary structure elements α-helices (cylinders) and β-strands (arrows) are also indicated above the sequences.

**FIGURE S2** Showing results of polyacrylamide gel electrophoresis for *Ab*Pth.

Lane A: molecular weight markers, 116.0 kDa - β-galactosidase, 66.2 kDa - bovine serum albumin, 45.0 kDa - ovalbumin, 35.0 kDa - lactate dehydrogenase, 25.0 kDa - ribonuclease, 18.4 kDa - β-lactoglobulin and 14.4 kDa - lysozyme; Lane B: purified protein, *Ab*Pth.

**FIGURE S3** Showing electron densities for the C-terminal segment, Pro181 – Ala193 (A) Fo-Fc map at 2.5 σ cut off and (B) 2Fo-Fc map at 1 σ cut off.

**FIGURE S4** Binding**s** of cytidine (A) and uridine (B) showing large conformational changes with side chains of Asn70, Asp98 and Asn116**.** The electron densities from the initial (Fo-Fc) map at 2.5 σ cut off.

β1

α1

β2

β3

β4

**10 20 30 40 50 60**

***Ab*Pth MSNISLIVGLGNPGSEYAQTRHNAGFWFVEQLADKYGITLKNDPKFHGISGRGNIEGHDV**

***Pa*Pth MTAVQLIVGLGNPGPEYDQTRHNAGALFVERLAHAQGVSLVADRKYFGLVGKFSHQGKDV**

***Ec*Pth --TIKLIVGLANPGAEYAATRHNAGAWFVDLLAERLRAPLREEAKFFGYTSRVTLGGEDV**

***Mt*Pth MAEPLLVVGLGNPGANYARTRHNLGFVVADLLAARLGAKFKAHKRSGAEVATGRSAGRSL**

***Ms*Pth MAEPLLVVGLGNPGPTYAKTRHNLGFMVADVLAGRIGSAFKVHKKSGAEVVTGRLAGTTV**

β4

α2

β5

β6

α3

**70 80 90 100 110 120**

***Ab*Pth RLLLPMTYMNRSGQSVVPFSKFYQIAPEAILIAHDELDMNPGVIRLKTGGGHGGHNGLRD**

***Pa*Pth RLLIPTTYMNRSGQSVAALAGFFRIAPDAILVAHDELDMPPGVAKLKTGGGHGGHNGLRD**

***Ec*Pth RLLVPTTFMNLSGKAVAAMASFFRINPDEILVAHDELDLPPGVAKFKLGGGHGGHNGLKD**

**MtPth VLAKPRCYMNESGRQIGPLAKFYSVAPANIIVIHDDLDLEFGRIRLKIGGGEGGHNGLRS**

**MsPth VLAKPRISMNESGRQVGPLAKFYSVPPQQIVVIHDELDIDFGRIRLKLGGGEGGHNGLRS**

α3

β7

α4

α5

**130 140 150 160 170**

**AbPth IVPHIG--PNFHRLRIGIGHPGSKERVSGHVLGKAPSNEQSLMDGAIDHALSKVKLLVQG**

**PaPth IIAQLGNQNSFHRLRLGIGHPGHSSLVSGYVLGRAPRSEQELLDTSIDFALGVLPEMLAG**

**EcPth IISKLGNNPNFHRLRIGIGHPGDKNKVVGFVLGKPPVSEQKLIDEAIDEAARCTEMWFTD**

**MtPth VVAALG-TKDFQRVRIGIGRPPGRKDPAAFVLENFTPAERAEVPTICEQAADATELLIEQ**

**MsPth VASALG-TKNFHRVRIGVGRPPGRKDPAAFVLENFTSAERAEVPTIVEQAADATELLIAQ**

α6

**180 190 Sequence identities**

**AbPth QVPQAMNQINAYKPA with *Ab*Pth (%)**

**PaPth DWTRAMQKLHSQKA- 58**

**EcPth GLTKATNRLHAFKAQ 53**

**MtPth GMEPAQNRVHAW--- 39**

**MsPth GLEPAQNTVHAW--- 40**

**Figure A1**


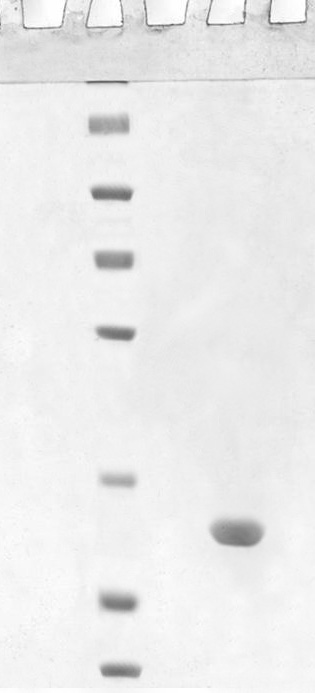


**MW**

**in kDa**

**116.0 document or the summary of an interesting point. You can position the text box anywhere in the document. Use the Text Box Tools tab to change the formatting of the pull quote text box.]**

**66.2 document or the summary of an interesting point. You can position the text box anywhere in the document. Use the Text Box Tools tab to change the formatting of the pull quote text box.]**

**45.0 document or the summary of an interesting point. You can position the text box anywhere in the document. Use the Text Box Tools tab to change the formatting of the pull quote text box.]**

**35.0 document or the summary of an interesting point. You can position the text box anywhere in the document. Use the Text Box Tools tab to change the formatting of the pull quote text box.]**

**25.0 document or the summary of an interesting point. You can position the text box anywhere in the document. Use the Text Box Tools tab to change the formatting of the pull quote text box.]**

**18.4 document or the summary of an interesting point. You can position the text box anywhere in the document. Use the Text Box Tools tab to change the formatting of the pull quote text box.]**

**14.4 document or the summary of an interesting point. You can position the text box anywhere in the document. Use the Text Box Tools tab to change the formatting of the pull quote text box.]**

**21.8 kDa**

**A B document or the summary of an interesting point. You can position the text box anywhere in the document. Use the Text Box Tools tab to change the formatting of the pull quote text box.]**

**Figure A2**

**Figure A3**

**Figure A4**
